# Corneal confocal microscopy identifies corneal nerve loss and increased Langerhans cells in presymptomatic carriers and patients with hereditary transthyretin amyloidosis

**DOI:** 10.1007/s00415-023-11689-z

**Published:** 2023-04-04

**Authors:** Andreas Thimm, Alexander Carpinteiro, Sara Oubari, Maria Papathanasiou, Lukas Kessler, Christoph Rischpler, Rayaz Ahmed Malik, Ken Herrmann, Hans Christian Reinhardt, Tienush Rassaf, Christoph Kleinschnitz, Tim Hagenacker, Mark Stettner

**Affiliations:** 1 Department of Neurology, University Hospital Essen, Hufelandstrasse 55, 45147 Essen, Germany; 2 Center for Translational Neuro- and Behavioral Scienes (C-TNBS), University Hospital Essen, Essen, Germany; 3 Department of Hematology and Stem Cell Transplantation, West German Cancer Center, University Hospital Essen, Essen, Germany; 4 Institute of Molecular Biology, University of Duisburg-Essen, Essen, Germany; 5 Department of Cardiology and Vascular Medicine, West German Heart and Vascular Center, University Hospital Essen, Essen, Germany; 6 Department of Nuclear Medicine, University Hospital Essen, Essen, Germany; 7 Institute of Cardiovascular Science, Faculty of Medical and Human Sciences, University of Manchester, Manchester, UK; 8 Weill Cornell Medicine-Qatar, Education City, Doha, Qatar

**Keywords:** Small fibre neuropathy, PADO concept, siRNA, Hereditary amyloidosis, Immune cells

## Abstract

**Background:**

Hereditary transthyretin amyloidosis (ATTRv amyloidosis) is a rare, but life-threatening protein misfolding disorder due to *TTR* gene mutations. Cardiomyopathy (ATTRv-CM) and polyneuropathy (ATTRv-PN) with early small nerve fibre involvement are the most common manifestations. Timely diagnosis and treatment initiation are key to limiting progression of disease. Corneal confocal microscopy (CCM) is a non-invasive method to quantify corneal small nerve fibres and immune cell infiltrates in vivo.

**Methods:**

This cross-sectional study investigated the utility of CCM in 20 patients with ATTRv amyloidosis (ATTRv-CM, n = 6; ATTRv-PN, n = 14) and presymptomatic carriers (n = 5) compared to 20 age- and sex-matched healthy controls. Corneal nerve fibre density, corneal nerve fibre length, corneal nerve branch density, and cell infiltrates were assessed.

**Results:**

Corneal nerve fibre density and nerve fibre length were significantly lower in patients with ATTRv amyloidosis compared to healthy controls regardless of the clinical phenotype (ATTRv-CM, ATTRv-PN) and corneal nerve fibre density was significantly lower in presymptomatic carriers. Immune cell infiltrates were only evident in patients with ATTRv amyloidosis, which correlated with reduced corneal nerve fibre density.

**Conclusions:**

CCM identifies small nerve fibre damage in presymptomatic carriers and symptomatic patients with ATTRv amyloidosis and may serve as a predictive surrogate marker to identify individuals at risk of developing symptomatic amyloidosis. Furthermore, increased corneal cell infiltration suggests an immune-mediated mechanism in the pathogenesis of amyloid neuropathy.

**Supplementary Information:**

The online version contains supplementary material available at 10.1007/s00415-023-11689-z.

## Introduction

Hereditary transthyretin (ATTRv) amyloidosis is a rare [1], but life-threatening autosomal-dominant systemic disorder characterised by extracellular deposition of misfolded transthyretin fibrils in various tissues [2]. Cardiomyopathy (ATTRv-CM) and progressive axonal polyneuropathy (ATTRv-PN) with early small fibre involvement and autonomic dysfunction are the most common manifestations [3]. To date, more than 120 amyloidogenic mutations of the *TTR* gene have been identified [4] and genetic heterogeneity is particularly high in non-endemic regions such as the USA and most of Europe except areas in Northern Portugal and Sweden [1, 5–7]. Phenotypic presentation as cardiac, neuropathic or mixed, partially depends on the underlying genotype [5, 8], but even in carriers of the same mutation, phenotypes may vary [9]. Age at disease onset ranges from 20 to 80 years, and penetrance is incomplete [10]. Genetic heterogeneity, clinical variability, inadequate diagnostic workup and low disease awareness frequently lead to a diagnostic delay [11], which is critical facing the fatal natural disease course. Untreated ATTRv amyloidosis leads to rapid disease progression, severe disability, and death within approximately 7–11 years [10, 12].

The approval of patisiran and vutrisiran, small interfering RNA drugs, and inotersen, an antisense oligonucleotide, which effectively supress transthyretin expression by degradation of TTR mRNA, have transformed the management of ATTRv-PN [13, 14]. Tafamidis, a stabilizer of the transthyretin tetramer is available for the treatment of isolated ATTRv-CM [15]. Given that timely treatment initiation improves outcomes in patients with ATTRv amyloidosis and amyloid deposition is considered to start before symptom onset, it remains an open question whether and when to start treatment in carriers, especially as treatments are burdensome and costly [10, 16–20]. A more precise assessment of underlying pathology may allow earlier intervention and assessment of disease progression.

Corneal confocal microscopy (CCM) is a rapid, reiterative, and non-invasive imaging technique to quantify small corneal nerve fibers in vivo [21]. It has proven diagnostic value in various peripheral neuropathies including diabetic [22–24], hereditary [25, 26] and immune-mediated neuropathies [27, 28]. It has also been used to assess immune cell infiltration in a number of neuropathies [29–31]. Differential origins of these cells has been discussed before. Professional antigen-presenting dendritic cells analogously to Langerhans cells of the skin [32–35] are present in the noninflamed corneal epithelium [36]. Since mature Langerhans cells feature dendrites whereas immature Langerhans cells lack dendrites, the differential cells detected can be discussed in this context [29, 37, 38].

However, data on CCM in patients with ATTRv amyloidosis are scarce, despite small fibre involvement being a frequent [39], early [40, 41] and prominent manifestation of ATTRv amyloidosis. This cross-sectional study assessed the utility of CCM as a tool for early diagnosis in a genetically heterogeneous and phenotypically variable cohort of patients with ATTRv amyloidosis and presymptomatic carriers.

## Patients and methods

### Patients

Investigations were performed in the Department of Neurology, University Hospital Essen between January 2020 and December 2021. Data from 20 patients with confirmed amyloidogenic *TTR* mutation and either isolated ATTRv-CM or ATTRv-PN with or without cardiac involvement, and 5 presymptomatic carriers (relatives of the aforementioned patients) were collected. All patients and carriers underwent transthoracic echocardiography, nerve conduction studies (NCS), detailed medical history and laboratory evaluation to exclude other causes of neuropathy e.g. exposure to alcohol or chemotherapy, or predisposing conditions such as diabetes, thyroid disorders or vitamin deficiencies. Carriers were considered presymptomatic if NCS, echocardiography, and clinical examination were normal. ATTRv amyloidosis was classsified as ATTRv-CM if there was an abnormality on transthoracic echocardiography, consistent with amyloid cardiomyopathy, but NCS and neurological examination were normal. Patients with neurological and NCS abnormality were classified as ATTRv-PN, with or without cardiomyopathy.

A reference group of 20 age- and sex-matched healthy individuals who had undergone clinical, neurological and neurophysiological testing and laboratory testing to exclude neuropathy was recruited from the University of Manchester, United Kingdom.

No participant had a known ophthalmologic disease or ocular symptoms at the time of the investigation, as these were exclusion criteria.

### Corneal confocal microscopy

**Fig. 1:**
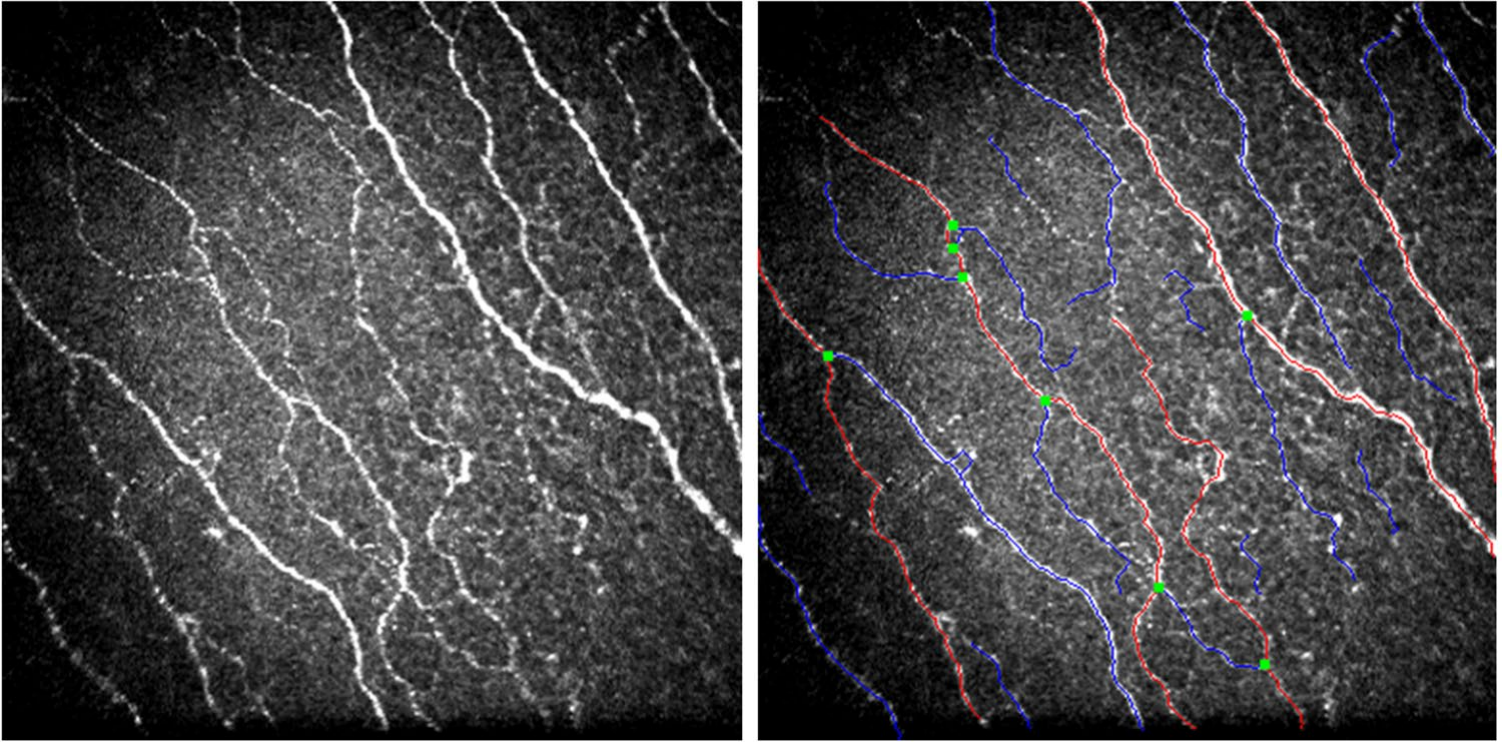
Representative image of corneal nerve fibres (left panel) and illustration of automated analysis of nerve fibre parameters (right panel) using ACCMetrics Image Analysis tool v1.1, University of Manchester, UK. Major nerves (red lines), axon collaterals (blue lines), and nerve branches (green dots)

**Fig. 2:**
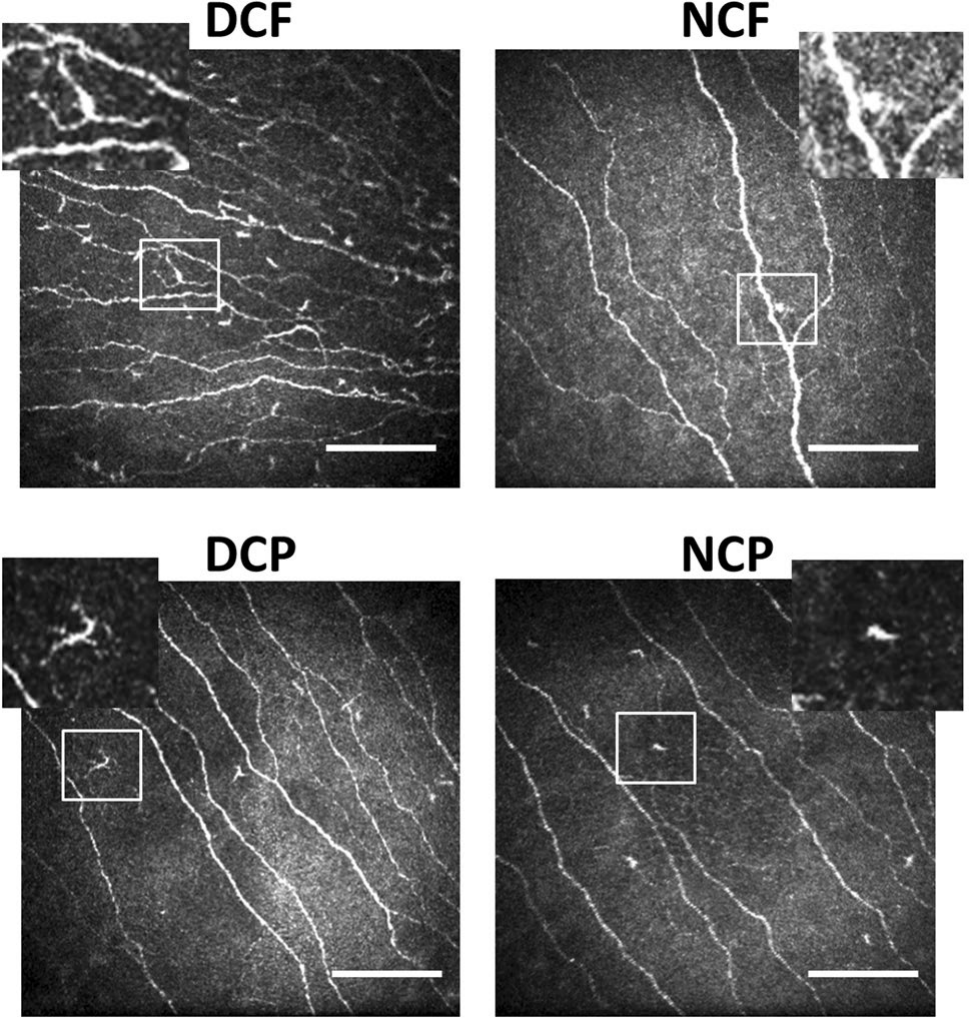
Representative images of the corneal cell subtypes identified according to their morphology and localisation. Dendritic cell with fibre contact (DCF, upper left panel), dendritic cell in the periphery without fibre contact (DCP, lower left panel), non-dendritic cell with fibre contact (NCF, upper right panel), and non-dendritic cell in the periphery without fibre contact (NCP, lower right panel). Scale bars = 100 µm

CCM was performed using a Heidelberg Retina Tomograph (HRT III, Rostock Cornea Module, Heidelberg Engineering, Heidelberg, Germany). A local anaesthetic (0.4% benoxinate hydrochloride) was administered immediately before the examination. Viscotears liquid gel was applied to the eye for lubrication and to establish a thin gel bridge between the corneal surface and a sterile, single-use lens cap. Several scan cycles of the entire depth of the cornea were carried out focusing on the sub-basal nerve plexus at the center of the cornea. The integrity of the corneal surface was visually confirmed and at least six images per patient meeting accepted quality criteria were analyzed [42]. Well-established software (ACCMetrics Image Analysis tool v1.1, University of Manchester, UK) was used for automated quantification of corneal nerve fibre density (CNFD, major nerves/mm^2^), corneal nerve fibre length (CNFL, mm/mm^2^), and corneal nerve branch density (CNBD, major branches/mm^2^, see Fig. 1). Corneal cells were counted manually by an independent investigator in a blinded fashion using ImageJ software (version 1.41, National Institutes of Health, Bethesda, Maryland, USA) and were classified according to their morphology, i.e. presence or absence of dendritic cell extensions, and their location relative to the corneal nerve fibres, as previously described [27]. Four subtypes, dendritic cells with fibre contact (DCF), dendritic cells in the periphery without fibre contact (DCP), non-dendritic cells with fibre contact (NCF), and non-dendritic cells in the periphery without fibre contact (NCP) were identified (see Fig. 2).

### Statistical analysis

Statistical analyses were performed using GraphPad Prism (version 9.1.0 for Windows, GraphPad Software, San Diego, California, USA). All data are presented as the mean, standard error of the mean, and *p* values. Differences in cell and nerve fibre parameters between patients with ATTRv-CM, ATTRv-PN and healthy controls and between ATTRv amyloidosis patients, presymptomatic carriers and healthy controls were assessed using a Kruskal–Wallis test and Dunn’s test as post hoc analysis. *P* values < 0.05 were considered to be statistically significant. Correlations were calculated using Spearman’s rank correlation coefficient.

## Results

Twenty patients with ATTRv amyloidosis (15 male, 5 female, mean age 64.1 ± 2.9 years) were compared to 20 healthy controls (15 male, 5 female, mean age 62.7 ± 1.7 years) and 5 presymptomatic carriers (2 male, 3 female, mean age 40.4 ± 2.8 years; for individual CCM data see Online Resource 1). Six patients were classified as ATTRv-CM patients (3 male, 3 female, mean age 67.7 ± 5.8 years), and 14 as ATTRv-PN patients (12 male, 2 female, mean age 62.9 ± 3.1 years). Patients and presymptomatic carriers were genetically heterogeneous with a wide variety of *TTR* mutations. Small fibre-mediated symptoms such as neuropathic pain and autonomic dysfunction were frequent in patients with symptomatic ATTRv amyloidosis. For detailed demographic and clinical characteristics see Table 1 and for NCS of the ATTRv-PN patients, see Online Resource 2.

**Table 1:** Characteristics of patients with ATTRv amyloidosis and presymptomatic carriers

	Symptomatic ATTRv amyloidosis	Presymptomatic carriers
Age (mean ± SEM, years)	64.1 ± 2.9	40.4 ± 2.8
Sex	15 male, 5 female	2 male, 3 female
Phenotype	6 ATTRv-CM, 14 ATTRv-PN	–
*TTR* mutation (number of individuals)		
p.Val50Met		
p.Ile88Leu	6	2
p.Leu78His	3	–
p.Arg54Gly	2	1
p.Ala65Thr	1	–
p.Ala65Val	1	–
p.Ser43Asn	1	–
p.Ile127Val	1	–
p.Val114Ala	1	1
p.Glu81Lys	1	1
p.Phe84Leu	1	–
p.Val142Ile	1	–
	1	–
Coutinho stages (ATTRv-PN), count (percentage)	Stage 1: 11 (78.6%) Stage 2: 3 (21.4%)	Stage 0: 100%
Years from symptom onset of index patient (mean ± SEM, years)	–	19.8 ± 5.6
Neuropathic pain, count (percentage)	12 (60.0%)	–
Autonomic symptoms, count (percentage)	10 (50.0%)	–
Orthostatic hypotension	6 (30.0%)	
Diarrhea/constipation	6 (30.0%)	
Erectile dysfunction	3 (15.0%)	
Bladder dysfunction	3 (15.0%)	
Treatment at the time of examination, count (percentage)		
Tafamidis	8 (40.0%)	–
Patisiran	10 (50.0%)	–
Inotersen	2 (10.0%)	–

**Fig. 3:**
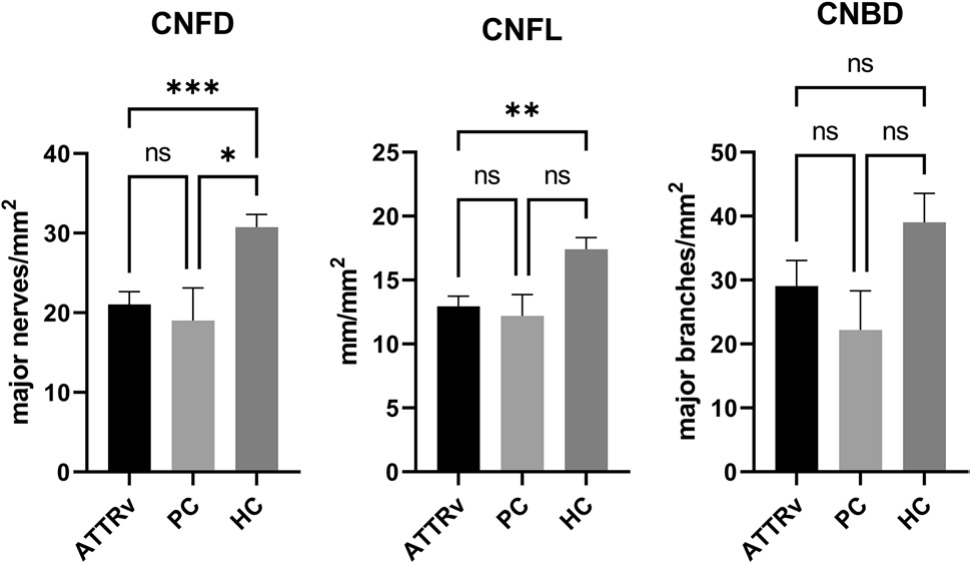
Corneal nerve fibre parameters in patients with ATTRv amyloidosis (ATTRv, black), presymptomatic carriers (PC, light grey), and healthy controls (HC, dark grey). Corneal nerve fibre density (CNFD), corneal nerve fibre length (CNFL), and corneal nerve branch density (CNBD) are displayed as Mean ± SEM, *p < 0.05, **p < 0.01. ***p < 0.001, ns, not significant

CNFD (21.05 ± 1.59 vs. 30.75 ± 1.58 fibres/mm^2^, p = 0.0003) and CNFL (12.95 ± 0.79 vs. 17.40 ± 0.93 mm/mm^2^, p = 0.0032) were significantly lower (Fig. 1), whilst CNBD did not differ significantly (29.05 ± 4.03 vs. 39.02 ± 4.55 branches/mm^2^, p = 0.119) in patients with ATTRv amyloidosis compared to healthy controls. In case of a CNFD below a threshold of 24 fibres/mm^2^ (two standard deviations below the mean of the reference group) the relative risk of symptomatic amyloidosis instead of being presymptomatic/healthy was 3,75 (15 out of 20 participants with CNFD < 24 fibres/mm^2^ and only 5 out of 25 participants with CNFD ≥ 24 fibres/mm^2^ were patients with symptomatic ATTRv amyloidosis). In presymptomatic carriers, CNFD was significantly lower compared to healthy controls (19.00 ± 4.11 vs. 30.75 ± 1.58 fibres/mm^2^, p = 0.025) and was comparable to patients with symptomatic amyloidosis (Fig. 3).

**Fig. 4:**
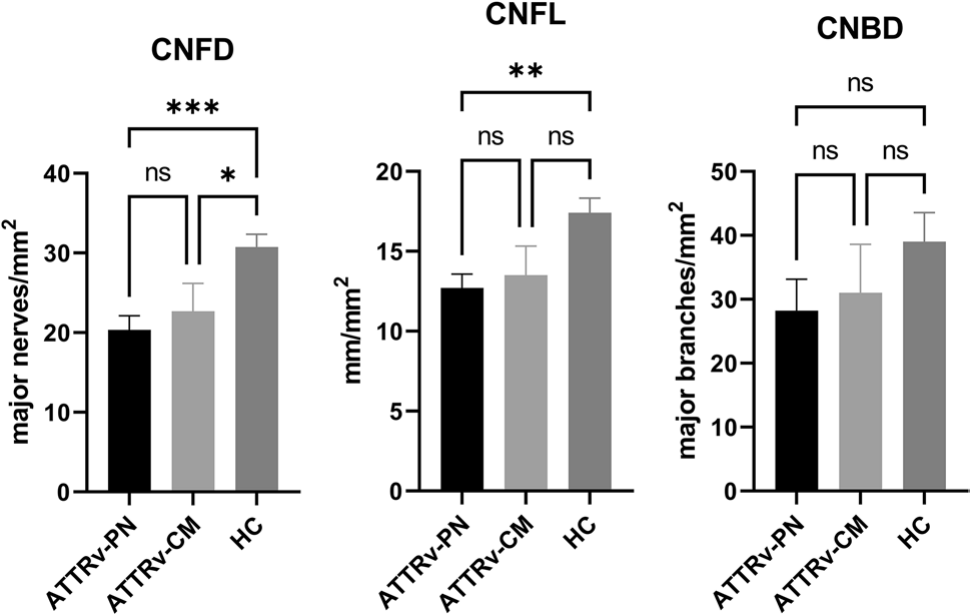
Subgroup analysis of corneal nerve fibre parameters in ATTRv patients with peripheral neuropathy (ATTRv-PN, black), cardiomyopathy (ATTRv-CM, light grey), and healthy controls (HC). Corneal nerve fibre density (CNFD), corneal nerve fibre length (CNFL), and corneal nerve branch density (CNBD) are displayed as mean ± SEM, *p < 0.05, **p < 0.01. ***p < 0.001, ns, not significant

In patients with ATTRv-PN, CNFD (20.36 ± 1.77 vs. 30.75 ± 1.58 fibres/mm^2^, p < 0.001) and CNFL (12.71 ± 0.87 vs. 17.40 ± 0.93 mm/mm^2^, p = 0.008, Fig. 4) were significantly lower compared to controls. In patients with ATTRv-CM, CNFD (22.67 ± 3.51 vs. 30.75 ± 1.58 fibres/mm^2^, p = 0.038, Fig. 4), but not CNFL was significantly reduced compared to healthy controls. CNBD did not differ from healthy controls in both ATTRv amyloidosis phenotypes.

**Fig. 5:**
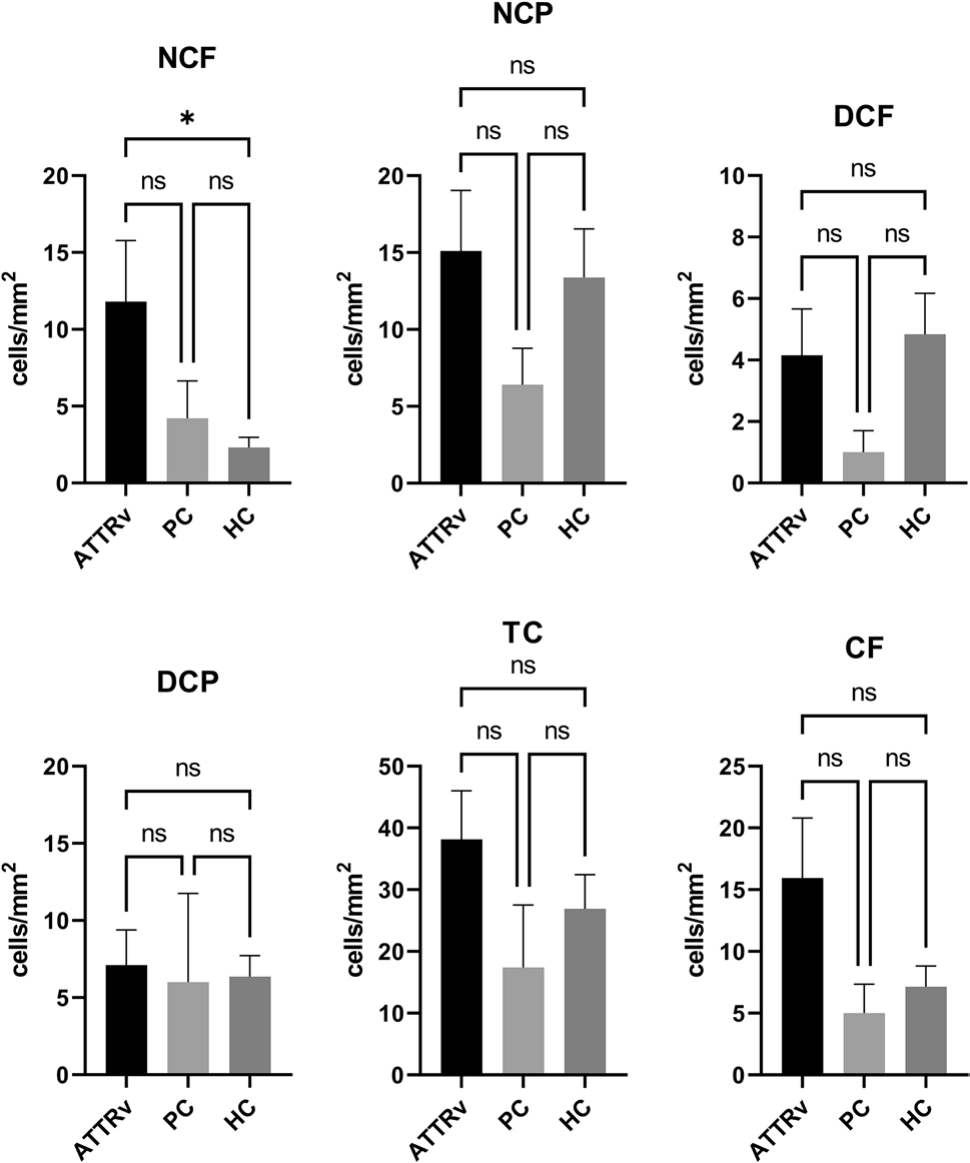
Corneal cell counts in patients with ATTRv amyloidosis (ATTRv, black), presymptomatic carriers (PC, light grey), and healthy controls (HC, dark grey)

In relation to corneal immune cell infiltration, NCF was significantly higher in ATTRv patients compared to healthy controls (11.80 ± 3.98 vs. 2.30 ± 0.68 cells/mm^2^, p = 0.021), but not in presymptomatic carriers (4.20 ± 2.43 vs. 2.30 ± 0.68 cells/mm^2^, p > 0.999). Total cell count (TC), DCF, DCP, and NCP did not differ between ATTRv patients and healthy controls (Fig. 5).

The total cell count (TC), cell counts of dendritic cells with fibre contact (DCF), dendritic cells in the periphery (DCP), non-dendritic cells with fibre contact (NCF), non-dendritic cells in the periphery (NCP), and all cells with fibre contact regardless of their morphology (CF) are displayed as mean ± SEM, *p < 0.05, **p < 0.01. ns, not significant.

**Fig. 6:**
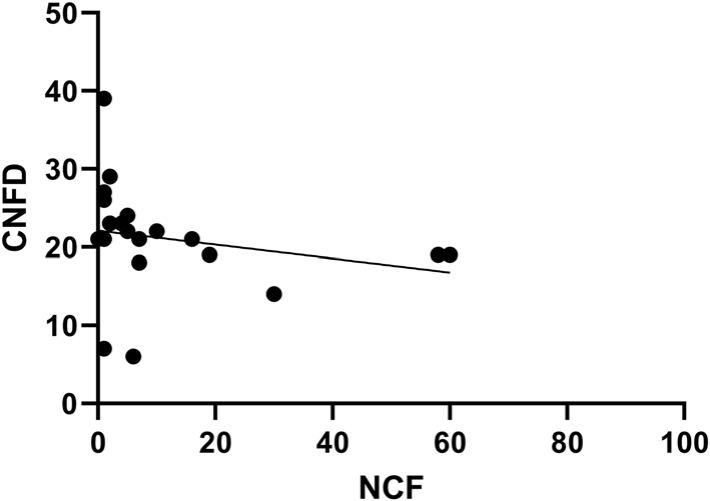
Correlation of non-dendritic cells with fibre contact (NCF) with corneal nerve fibre density (CNFD) in patients with ATTRv amyloidosis

**Fig. 7:**
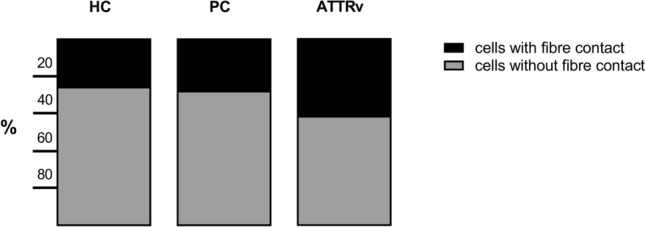
Proportion of cells with (black) and without (grey) fibre contact in patients with ATTRv amyloidosis (left), presymptomatic carriers (middle), and healthy controls (right)

There were no significant differences between ATTRv-CM and ATTRv-PN (data not shown). NCF (r = − 0.50, p = 0.026), but not DCF, correlated inversely with CNFD (Fig. 6) in patients with ATTRv amyloidosis. The proportion of corneal cells with fibre contact was higher in patients with ATTRv amyloidosis (44%), but only marginally in presymptomatic carriers (28%) compared to healthy controls (26%) (Fig. 7).

## Discussion and conclusions

This study has utilized CCM to identify a significant reduction of corneal nerve fibres in patients with ATTRv amyloidosis regardless of clinical phenotype or *TTR* mutation and in presymptomatic carriers decades before reaching the age of disease onset in index patients.

Early diagnosis and treatment are key to the management of patients with ATTRv amyloidosis. However, defining disease onset in oligosymptomatic patients can be challenging and monitoring algorithms in presymptomatic carriers remain a matter of debate [43]. Current expert consensus recommends annual follow-up of carriers commencing ten years prior to the predicted age of disease onset (PADO), depending on the *TTR* mutation, age of onset in the individual and family members with amyloidosis [44]. However, cases of early- and late-onset ATTRv amyloidosis can be found within the same family or genotype [45] and amyloid deposition is thought to precede symptom onset by several years similar to other protein misfolding disorders [2]. There is an urgent need for reliable, reproducible and non-invasive biomarkers of disease onset and progression.

Our findings suggest that CCM may identify the onset of subclinical amyloid neuropathy in presymptomatic carriers, as there was a striking loss of nerve fibres in carriers who were approximately twenty years younger than their relatives with apparent amyloidosis at disease onset. CCM may therefore qualify as a predictive surrogate marker to identify presymptomatic carriers at risk of developing ATTR related neuropathy. The fact that CNBD was not altered in ATTRv amyloidosis patients, whilst CNFD and CNFL were reduced, might be explained by a compensatory axonal regeneration at early stages of the disease.

Additionally, corneal immune cell infiltration could be used to differentiate between presymptomatic carriers and symptomatic patients, as significant corneal immune cell infiltration, namely an increase in NCF, only occurred in the latter. The corneal immune cells we classified as non-dendritic cells according to morphological criteria have been discussed before to be resident immature Langerhans cells. Previously, it has been shown that the number, proportion and distribution of corneal Langerhans cell subtypes depends on various factors such as the ocular microenvironment [46], the corneal region since mature Langerhans cells are predominantly present in the periphery [34, 37], and even diurnal variations [47] in healthy individuals. Nevertheless, the increase in cells with fibre contact in this study, albeit non-dendritic ones, and their correlation with reduced nerve fibre density suggest an immune mediated [27, 28] mechanism of amyloid neuropathy or at least secondary inflammatory processes. Although an upregulation of pro-inflammatory cytokines [48] and an induction of inflammatory responses through macrophage activation in the sural nerve [49] have been shown previously in patients with amyloidosis, current research into the disease mechanisms has focused on mechanical compression of nerve fibres, ischemia due to perivascular amyloid deposition, and toxic effects of non-fibrillar aggregates [50–52]. Our data are supported by a recent study showing a clustering of immature Langerhans cells at the inferior whorl in ATTRv amyloidosis patients [53]. Finally, it remains to be determined, whether CCM can help to identify secondary inflammatory processes as a therapeutic target in patients with ATTRv amyloidosis.

Two small cohort studies have previously utilized CCM in patients with ATTRv amyloidosis. Rousseau et al. [54] found reduced CNFL in 15 patients with ATTRv amyloidosis (10 patients with p.Val50Met) which is consistent with our results and was associated with more severe autonomic dysfunction and reduced intraepidermal nerve fibre density. There was no change in CNFL in 2 presymptomatic carriers included, also consistent with our data. However, this study did not examine other nerve fibre parameters than CNFL. A recent study from China [44] showed a reduction in corneal nerve fibre length at the inferior whorl in patients with ATTRv amyloidosis, which correlated with disease severity defined by clinical disease stages. However, they included 5 presymptomatic carriers and only 10 patients with ATTRv amyloidosis with the same mutation (p.Ala117Ser) and the healthy controls were considerably younger, potentially affecting CNFD which was indeed much higher than in our controls, and they did not investigate clinically defined subgroups as we did.

A relevant limitation of this study and the mentioned studies is the small cohort of investigated patients and especially carriers. This is a result of ATTRv amyloidosis being a very rare disease outside endemic areas. Furthermore, management of presymptomatic carriers requires careful genetic counseling and many individuals at risk avoid molecular genetic testing expecting socioeconomic disadvantages associated with a positive result.

In conclusion, CCM, a non-invasive ophthalmic technique enables the identification of small fibre involvement across diverse genotypes and clinical phenotypes, especially early in the course of ATTRv amyloidosis. It also sheds new light on possible immune-mediated mechanisms of small fibre neuropathy in ATTRv amyloidosis. CCM could be a sensitive diagnostic tool to enable early diagnosis and to monitor mutation carriers, even earlier than currently recommended following the PADO concept. Longitudinal studies are warranted to assess the predictive value of CCM in the development and progression of different ATTRv phenotypes.

## Supplementary Information

Below is the link to the electronic supplementary material.

Supplementary file1 (DOCX 13 KB)

Supplementary file2 (DOCX 15 KB)

## Data Availability

Data are available from the corresponding author upon reasonable request by any qualified investigator.
