# Supplementary material for: Corneal confocal microscopy identifies corneal nerve loss and increased Langerhans cells in presymptomatic carriers and patients with hereditary transthyretin amyloidosis
Source: J Neurol. 2023 Apr 4;270(7):3483–91. doi: 10.1007/s00415-023-11689-z (PMC10267010; doi:10.1007/s00415-023-11689-z)
Supplement: Supplementary file 1 — Supplementary file1 (DOCX 13 KB) [file 415_2023_11689_MOESM1_ESM.docx]

**Online Resource 1.** CCM results in individual presymptomatic carriers

|  | *TTR* mutation | CNFD (fibres/mm^2^) | CNFL (mm/mm^2^) | CNBD (branches/mm^2^) | DCF (cells/mm^2^) | DCP (cells/mm^2^) | NCF (cells/mm^2^) | NCP (cells/mm^2^) |
| --- | --- | --- | --- | --- | --- | --- | --- | --- |
| PC1 | p.Ile127Val | 12 | 15 | 36 | 0 | 29 | 13 | 15 |
| PC2 | p.Val114Ala | 22 | 10 | 15 | 1 | 0 | 6 | 8 |
| PC3 | p.Leu78His | 25 | 13 | 29 | 0 | 0 | 1 | 2 |
| PC4 | p.Val50Met | 29 | 16 | 29 | 3 | 0 | 1 | 4 |
| PC5 | p.Val50Met | 7 | 7 | 2 | 0 | 1 | 0 | 3 |

**PC1-5 = presymptomatic carrier 1-5; CNFD = corneal nerve fibre density; CNFL = corneal nerve fibre length; CNBD = corneal nerve branch density; DCF = dendritic cells with fibre contact; DCP = dendritic cells in the periphery; NCF = non-dendritic cells with fibre contact; NCP = non-dendritic cells in the periphery.**
