# Supplementary material for: Corneal confocal microscopy identifies corneal nerve loss and increased Langerhans cells in presymptomatic carriers and patients with hereditary transthyretin amyloidosis
Source: J Neurol. 2023 Apr 4;270(7):3483–91. doi: 10.1007/s00415-023-11689-z (PMC10267010; doi:10.1007/s00415-023-11689-z)
Supplement: Supplementary file 2 — Supplementary file2 (DOCX 15 KB) [file 415_2023_11689_MOESM2_ESM.docx]

**Online Resource 1.** NCS results in patients with ATTRv-PN

|  | Ulnar nerve, right | | | Tibial nerve, right | | | Tibial nerve, left | | | Sural nerve, right | | Sural nerve, left | |
| --- | --- | --- | --- | --- | --- | --- | --- | --- | --- | --- | --- | --- | --- |
|  | dML (ms) | CMAP/SNAP (mV/µV) | NCV (motor/sensory,  m/s) | dML (ms) | CMAP (mV) | NCV (m/s) | dML (ms) | CMAP (mV) | NCV  (m/s) | SNAP (µV) | NCV (m/s) | SNAP  (µV) | NCV  (m/s) |
| P1 | 2.62 | 8.7/19.62 | 59.9/62.2 | 5.10 | **0.85** | 42.2 | **5.85** | **0.50** | 42.8 | **NS** | **NS** | **2.90** | 50.0 |
| P2 | 2.44 | 8.5/12.0 | 51.4/42.4 | 3.68 | **0.53** | 42.9 | 4.55 | **2.6** | 41.3 | **NS** | **NS** | **4.0** | 51.9 |
| P3 | 2.23 | 6.1/6.7 | 53.0/59.1 | **NS** | **NS** | **NS** | **5.38** | **0.44** | **30.1** | **NS** | **NS** | **NS** | **NS** |
| P4 | 3.23 | 5.9/**4.6** | 66.2/**29.4** | **NS** | **NS** | **NS** | **6.38** | **0.15** | **33.9** | **NS** | **NS** | **NS** | **NS** |
| P5 | 3.29 | **2.4/NS** | **56.3/NS** | **NS** | **NS** | **NS** | **NS** | **NS** | **NS** | **NS** | **NS** | **NS** | **NS** |
| P6 | 2.58 | 7.2/13.8 | 51.0/59.8 | 4.58 | **1.2** | 40.9 | 4.93 | **2.9** | 41.5 | 8.0 | 52.7 | **3.3** | 47.8 |
| P7 | **4.21** | **2.9/NS** | **46.7/NS** | **8.73** | **0.22** | **26.5** | **NS** | **NS** | **NS** | **NS** | **NS** | **NS** | **NS** |
| P8 | 2.51 | 8.2/21.4 | 51.1/**41.2** | 4.41 | **1.76** | **39.4** | 3.24 | 6.6 | **39.4** | 5.8 | 41.8 | 7.8 | 42.1 |
| P9 | 2.51 | **2.3/NS** | 66.3/**NS** | **NS** | **NS** | **NS** | **NS** | **NS** | **NS** | **NS** | **NS** | **NS** | **NS** |
| P10 | 3.04 | 5.1/**4.1** | 51.1/49.1 | **NS** | **NS** | **NS** | **NS** | **NS** | **NS** | **NS** | **NS** | **NS** | **NS** |
| P11 | 2.83 | 7.6/22.2 | 54.1/51.1 | 4.18 | **0.32** | **41.0** | 4.77 | **0.44** | 41.3 | **4.4** | 41.7 | **4.5** | 43.8 |
| P12 | 3.21 | 8.2/18.2 | **49.1/36.0** | **NS** | **NS** | **NS** | **NS** | **NS** | **NS** | **NS** | **NS** | **NS** | **NS** |
| P13 | 2.51 | 6.8/9.1 | 51.2/**44.4** | 3.86 | **0.85** | **36.3** | 3.69 | **1.87** | **33.6** | **4.9** | 48.0 | 5.4 | **31.3** |
| P14 | 2.70 | **1.9/3.4** | **39.7/36.1** | **NS** | **NS** | **NS** | **NS** | **NS** | **NS** | **NS** | **NS** | **NS** | **NS** |
| **Reference values** | **<3.3** | **>4.0/>5.8** | **>50.6/>44.6** | **<5.2** | **>5.0** | **>40.6** | **<5.2** | **>5.0** | **>40.6** | **>4.9** | **>41.3** | **>4.9** | **>41.3** |

**Large fibre neuropathy (grey shaded) was defined by at least three affected nerves (pathological values in bold). dML, distal motor latency; CMAP, compound motor action potential; SNAP, sensory nerve action potential; NCV, nerve conduction velocity; P1-14, patient 1-14; NS, no measurable signa**
